# MOtoNMS: A MATLAB toolbox to process motion data for neuromusculoskeletal modeling and simulation

**DOI:** 10.1186/s13029-015-0044-4

**Published:** 2015-11-16

**Authors:** Alice Mantoan, Claudio Pizzolato, Massimo Sartori, Zimi Sawacha, Claudio Cobelli, Monica Reggiani

**Affiliations:** Department of Management and Engineering, University of Padova, Stradella San Nicola, 3, Vicenza, 36100 Italy; Centre for Musculoskeletal Research, Griffith University, Gold Coast campus, Gold Coast QLD, 4222 Australia; Department of Neurorehabilitation Engineering, University Medical Center Goettingen, Georg-August University, Von-Siebold-Str., 6, Goettingen, 37075 Germany; Department of Information Engineering, University of Padova, Via Gradenigo, 6/b, Padova, 35131 Italy

**Keywords:** Neuromusculoskeletal modeling, Motion data, Data processing, OpenSim, C3D

## Abstract

**Background:**

Neuromusculoskeletal modeling and simulation enable investigation of the neuromusculoskeletal system and its role in human movement dynamics. These methods are progressively introduced into daily clinical practice. However, a major factor limiting this translation is the lack of robust tools for the pre-processing of experimental movement data for their use in neuromusculoskeletal modeling software.

**Results:**

This paper presents MOtoNMS (matlab MOtion data elaboration TOolbox for NeuroMusculoSkeletal applications), a toolbox freely available to the community, that aims to fill this lack. MOtoNMS processes experimental data from different motion analysis devices and generates input data for neuromusculoskeletal modeling and simulation software, such as OpenSim and CEINMS (Calibrated EMG-Informed NMS Modelling Toolbox). MOtoNMS implements commonly required processing steps and its generic architecture simplifies the integration of new user-defined processing components. MOtoNMS allows users to setup their laboratory configurations and processing procedures through user-friendly graphical interfaces, without requiring advanced computer skills. Finally, configuration choices can be stored enabling the full reproduction of the processing steps. MOtoNMS is released under GNU General Public License and it is available at the SimTK website and from the GitHub repository. Motion data collected at four institutions demonstrate that, despite differences in laboratory instrumentation and procedures, MOtoNMS succeeds in processing data and producing consistent inputs for OpenSim and CEINMS.

**Conclusions:**

MOtoNMS fills the gap between motion analysis and neuromusculoskeletal modeling and simulation. Its support to several devices, a complete implementation of the pre-processing procedures, its simple extensibility, the available user interfaces, and its free availability can boost the translation of neuromusculoskeletal methods in daily and clinical practice.

## Background

Neuromusculoskeletal modeling and dynamics simulation have recently emerged as powerful tools to establish the causal relation between the neuromusculoskeletal system function and the observed movement. They estimate human internal variables, such as neural signals and muscle dynamics, that could not be derived by experimental measures and conventional motion analysis [1–5]. This provides a key contribution to fully understand human locomotion in healthy subjects and to establish a scientific basis for rehabilitation treatment of pathological movements [2, 5, 6].

In the latest years, several software tools (e.g., SIMM, AnyBody, OpenSim, MSMS) were released to automate and facilitate the complex and time-consuming process of modeling and simulate the movement of musculoskeletal systems [7–10]. Among them, the freely available OpenSim software has seen a widespread adoption with a growing network of research applications [4, 11–14].

Regardless the applications and the final objective of the study, these software tools require as input the simultaneous recordings of heterogeneous motion data acquired with different devices: three-dimensional marker trajectories, foot ground reaction forces (GRFs), and, often, surface electromyography (EMG). Before the recorded raw data can actually be used as input for the simulation softwares, several pre-processing steps are required depending on the objective of the study [15, 16]. Among them, filtering is usually performed and is one of the most critical [17, 18]. In addition, simpler steps as transformations among coordinate systems of the acquisition devices and the musculoskeletal modeling software still require to be carefully defined. Finally, the integrated and pre-processed motion data must be stored using the file format of the chosen simulation software.

While mature tools are available for the analysis of biomechanical data [19], there is still a lack of a robust tool for the pre-processing of experimental recorded data for optimal integration in neuromusculoskeletal modeling and simulation software. This represents a major factor limiting the translation of neuromusculoskeletal studies into daily practice, as highlighted by several researchers [13, 20, 21].

The main cause holding back the development of such a tool is probably the large number of commercially available motion analysis devices and proprietary softwares [13, 20, 22]. It is therefore difficult to handle all data seamlessly and with unified procedures. As a recognized problem, the biomechanics community proposed a standard file format (C3D – Coordinate 3D, [23]) to store all the heterogenous motion data: raw coordinate of 3D points, raw analog data from synchronized devices, force plates calibration, analog channels configuration, sample rates, and quantities computed by the acquisition software (joint angle, joint moment, joint power, …).

Despite the maturity of C3D, its use is still limited. Most of the companies provide acquisition systems that record information using different file formats and proprietary software tools that mainly process data with their own format. The consequence is that researchers develop a proliferation of custom tools and codes that perform similar processing pipeline, but might differ for the input data format and for the use of procedures and proprietary software specific to an acquisition system. As the latter are usually not openly available, it becomes difficult to reproduce the same data processing procedures in a consistent and repeatable way across different laboratories [20, 24].

Over the last years, the problem escalated as emerging biomechanics research challenges require multidisciplinary knowledge stimulating multicenter collaborations [25, 26]. Thus, the definition of shared and standard procedures for biomechanical data collection, management, and processing is increasingly required [20, 24].

This work presents MOtoNMS (matlab MOtion data elaboration TOolbox for NeuroMusculoSkeletal applications), a software toolbox that directly addresses this problem. MOtoNMS is an open source software [27] that has been already successfully used to process and share data from different laboratories, each one with its own gait analysis instrumentation and methodologies, for their use in neuromusculoskeletal analyses and applications.

The procedures implemented in MOtoNMS include: (i) computation of centers of pressure and torques for the most commonly available force platforms (types 1 to 4, including Bertec, AMTI, and Kistler); (ii) transformation of data between different coordinate systems; (iii) EMG filtering, maximum EMG peak computation, and EMG normalization; (iv) different procedures for gait events detection; (v) joint centers computation methods for hip, knee, ankle, elbow, shoulder, and wrist; (vi) support for OpenSim file formats and possibility to configure new output formats.

While MOtoNMS already provides a library of modules for the most commonly required steps, its architecture is designed to be open to new contributions in instrumentations, protocols, and methodologies. The choice of MATLAB, the most widespread language among biomechanists, goes also in the direction of simplifying the sharing of procedures within the community.

This paper describes the toolbox structure and modules, and then introduces the testing procedure. Finally, the paper points out MOtoNMS key features and main advantages. Motion data and results, freely available, show that MOtoNMS can handle experimental data collected in motion analysis laboratories with different setups and can process them to provide inputs for OpenSim [9] and CEINMS [28, 29]. The latter is a freely available neuromusculoskeletal software, developed by the authors’ research groups, that uses experimentally recorded EMG signals as estimates of the individual muscle recruitment strategies to predict muscle forces and joint moments [30].

## Methods

The MOtoNMS toolbox is implemented in MATLAB (The MathWorks, USA) and is intended to be accessible to a wide spectrum of users, from researchers to clinicians, who are interested in pre-processing experimental motion data to be used in neuromusculoskeletal simulations. The selection and setup of procedures is available through a set of graphical user interfaces, thus not requiring end-users to have advanced computer skills. Current MOtoNMS release works with MATLAB R2010b and later versions, and runs on the major operating systems (Windows, Linux, and MacOS X).

**Fig. 1:**
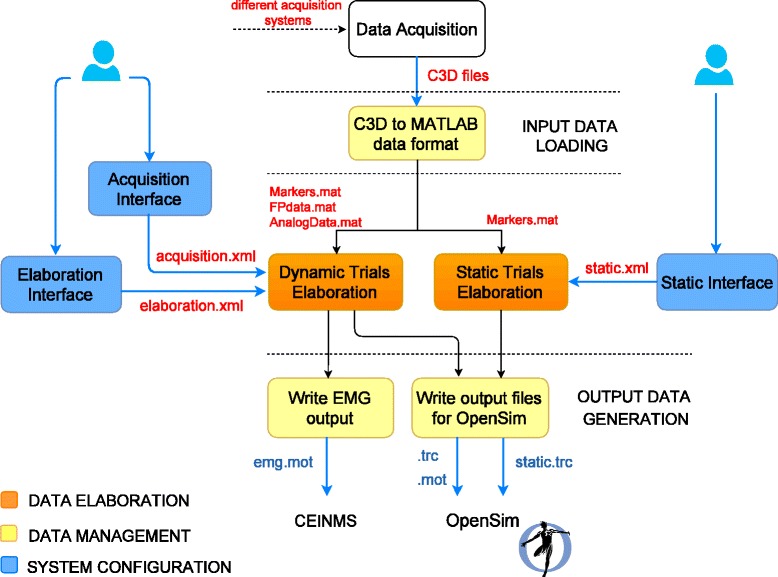
MOtoNMS overview schema. *Data Elaboration* is the toolbox core, processing data according to the user’s choices selected during the *System Configuration* steps. *Data Management* defines storing and management of input and output files

Figure 1 presents the toolbox organization. MOtoNMS comprises several blocks that are grouped in three main functional areas: *Data Elaboration*, with the procedures for the data processing pipeline, *Data Management*, responsible for the input data loading and the output data generation and storing, and *System Configuration*, supporting the user in the configuration of the elaboration through user friendly graphical interfaces. This structure, distributing independent modules with precise duties and well-defined input/output interfaces in three areas, simplifies the integration of other functionalities and algorithms.

### Data Elaboration

Data Elaboration is the toolbox core with the two blocks of Dynamic Trials Elaboration and Static Trials Elaboration. These are responsible for processing EMG, GRFs, and marker trajectories for dynamic and static trials.

#### Dynamic Trials Elaboration

**Fig. 2:**
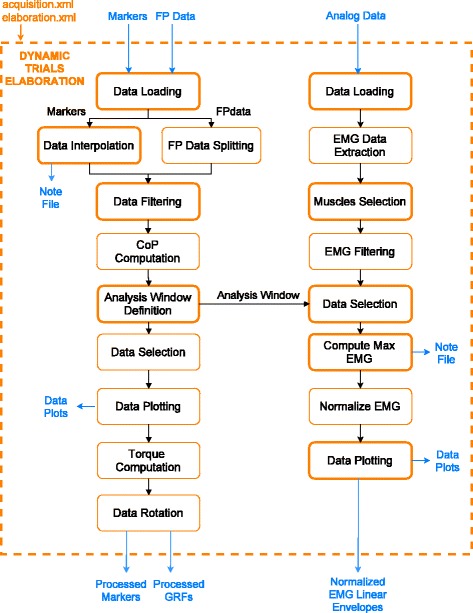
Dynamic Trials Elaboration. Flowchart of the Dynamic Trials Elaboration block. The user can customize this processing workflow by means of parameters defined in the *elaboration.xml* configuration file. Sub-blocks influenced by those parameters are emphasized with a bold line

This block (Fig. 2) handles motion data recorded from dynamic trials. It supports the different GRF data structures generated by the most common force plate (FP) types [31], with no constraints on the number and position of FPs in the laboratory. Depending on the FP type and its output, MOtoNMS correctly extracts raw force data, plate moments, and, when available, centers of pressure (CoP) [31]. For FP of type 3, total raw forces and moments are computed [32]. Three-dimensional marker trajectories undergo piecewise cubic interpolation when gaps caused by occlusions during the acquisition are automatically identified. Users can define the gap’s maximum size that will be interpolated. Choosing a value of zero results in no interpolation. A log file tracing the procedure is also available. Users can enable the filtering of pre-processed marker data and raw GRFs with a zero-lag second order low pass Butterworth filter at customizable cut-off frequencies. When CoPs are not directly provided by the FPs (types 2 to 4) [31], they are computed from filtered and thresholded forces and moments [32].

The analysis window definition sub-block (Fig. 2) allows selection of the data segments to be processed according to users choices. Frames of interest can be selected based on events, when available in the input C3D files. Alternatively, a thresholding algorithm based on GRF data is implemented for automatic detection of heel strike and toe off events [33]. Lastly, a manual selection of start and stop frames is also possible. Processed GRFs are then used to compute FP free torques [34] based on filtered forces, moments, and CoP for the selected frames. Finally, marker and GRF data are transformed from laboratory or FP reference systems to the global reference system of the selected musculoskeletal application, i.e. OpenSim. Required rotations depend on the laboratory setup described in the dedicated configuration file (“System Configuration” Section).

When available, raw EMG signals are processed by high-pass filtering, rectification, and low-pass filtering [28]. Resulting EMG linear envelopes are then normalized. For each muscle, the maximum EMG peak is identified by extracting the maximum instantaneous value from a set of trials selected by the user for the specific purpose. Those values are then logged in a text file. Other intermediate processing results (i.e., selected and processed EMG, filtered GRFs, CoPs, and moments within the analysis window) are also stored in dedicated folders, together with plots that facilitate their visual inspection.

#### Static Trials Elaboration

The objective of the Static Trials Elaboration block is to optimize data for the scaling of generic musculoskeletal models, which is essential to match an individual’s anthropometry [9]. Therefore it processes marker trajectories recorded during static standing trials and provides methods for the computation of subject-specific joint centers, which are usually recommended to improve the accuracy of the scaling procedure. This block is designed to accommodate different algorithms for the joint centers estimation. Users can include their own procedures for the joints of interest. Currently, MOtoNMS provides joint centers computation methods for hip, knee, ankle, elbow, shoulder, and wrist. Hip joint center is estimated through Harrington method [35], while the others are computed as the mid points between anatomical landmarks specified by the user.

### Data Management

Data Management (Fig. 1) deals with input and output data, supporting an easy integration of new file formats and inducing a clear and uniquely defined organization of the files. This is achieved also through a complete separation between Data Management and Data Elaboration.

#### Input data loading

Input data are extracted from C3D files and stored in MATLAB structures. This avoids continuous and computationally expensive access to C3D files. The extracted data include: marker trajectories, FP characteristics, GRFs, EMG signals, other data from analog channels, and events. Two implementations for data extraction are available: using C3Dserver software [23], limited to MATLAB 32 bit on Window platforms, or exploiting the Biomechanical Toolkit (BTK, [19]). Users can choose between the two alternatives according to the system requirements, with the second one enabling cross-platform execution.

The choice of supporting only C3D as input file format does not limit the usability of MOtoNMS. Indeed, being the standard for the representation of biomechanical data, usually acquisition systems (Vicon, Qualysis, BTS, MotionAnalysis, Codamotion, etc.) export synchronized data in the C3D file format.

#### Output data generation

The processed marker trajectories and GRFs are stored in.trc and.mot files (OpenSim file formats). The EMG linear envelopes are exported by default to.mot files (SIMM and OpenSim motion format), compatible also with the CEINMS toolbox [30]. Alternative file formats can be selected by the user, such as.sto (OpenSim storage) and text formats. The support of new file formats for other musculoskeletal modeling software requires the implementation of additional output blocks. These have only to store in the desired file formats the data already available from the processing phase, thus not introducing any change in the Data Elaboration step (Fig. 1).

#### Data storage structure

**Fig. 3:**
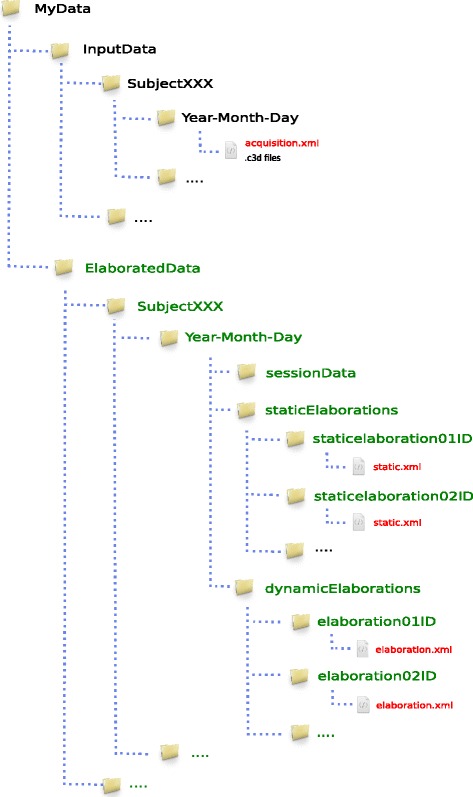
Data Folders Organization. Folders in black store input data. The picture presents the structure suggested by MOtoNMS authors: a folder for each subject that includes a set of directories, each one for a different acquisition session. All subjects must be grouped in a InputData folder. Red files are the configuration files, while green folders are for the output generated by the toolbox. These folders are automatically created and mirror the structure of the InputData folder. MOtoNMS reads C3D files and saves the extracted data in the sessionData subfolder. staticElaborations and dynamicElaborations subfolders include the output respectively of the Static Trials Elaboration and the Dynamic Trials Elaboration blocks. Finally, the results of multiple executions of these two parts, with different configurations for the same input data, are stored in different subfolders, each one named with an identifier chosen by the user through the graphical interface

MOtoNMS automatically generates output directories mirroring the structure of the data folders provided by the user. This relieves the user from manually creating the output folders and also results in a consistent structure, simplifying information retrieval. Albeit not mandatory, MOtoNMS authors encourage users to follow few simple suggestions in the organization of input experimental data, to foster the sharing of tools and results among research teams (Fig. 3).

### System Configuration

**Fig. 4:**
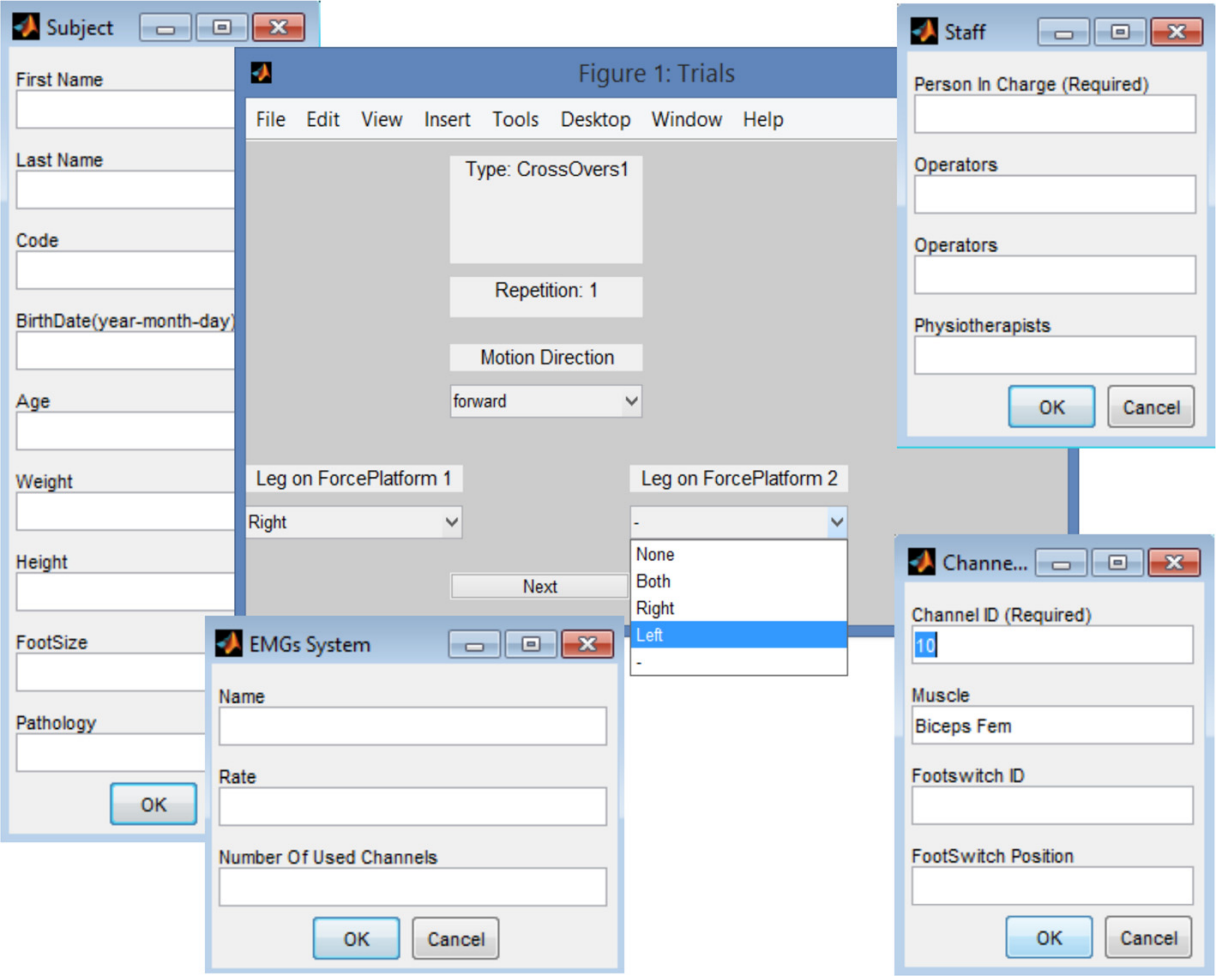
MOtoNMS GUI. Examples of user-friendly graphical MATLAB interfaces available in MOtoNMS for the configuration of the toolbox procedures (acquisition, elaboration, static configuration files)



The high configurability of MOtoNMS results in a high number of parameters. These are not set directly in the code as it would make the system hard to maintain. Instead, MOtoNMS can be fully configured through configuration files without modifying the underlying MATLAB code. Moreover, the use of configuration files guarantees the reproducibility of the data processing. Parameters are defined in three files: (1) *acquisition*, including information about the acquisition session (i.e., number of FP, coordinate system orientations, marker sets, and EMG setups), (2) *elaboration*, including parameters that univocally define the execution of the Dynamic Trials Elaboration block (i.e., selected trials, cut-off frequencies, markers list for output file, …, Lst. ??), and (3) *static*, including additional parameters for the elaboration of static trials (i.e., joint centers of interest). MOtoNMS stores a copy of the configuration files together with the output to keep a trace of performed elaborations [36]. The chosen language for these files is XML (eXtensible Markup Language), extremely suitable for parameter information encoding (Lst. ??). Syntax correctness of each file is guaranteed through the use of XML Schema Definition (XSD). MOtoNMS provides user-friendly MATLAB graphical interfaces that allow the user to handily configure the toolbox execution and automatically create the XML configuration files, ensuring their syntax correctness (Fig. 4). In addition, the configuration procedure has been designed to limit the required information to the one specific of the current experimental session. Those features that are common to several acquisition sessions (e.g., laboratory setup, marker and EMG protocols) are instead conveniently stored into XML files during the initial setup. These files can be selected from the GUI, so the user is not required to input all the included information at each new acquisition, thus resulting in an efficient system configuration procedure.

## Results

Data from four institutions were processed using MOtoNMS. The four gait laboratories are characterized by different instrumentations and setup (Table 1): (1) three motion capture systems: BTS, Vicon, and Qualysis; (2) three types of FPs [23], requiring different computation for plates moments and CoP; (3) four different setups for the global reference system, and FP positions and orientations along the walkway, resulting in different rotations from each FP reference system to the global one; (4) different configurations of analog channels; and (5) marker and EMG protocols dependent on each laboratory routine analysis.

**Table 1:** Characteristics of the laboratories testing MOtoNMS

Institution	Acquisition device	Global reference	Kinematic	Markers protocol	EMG device	Analog	Analog channels:
	(Hardware/Software)	system	sampling rate (Hz)			rate (Hz)	Output data
UNIPD	BTS Smart E		60	modified version of	BTS Pocket EMG	1020	1-6: FP1; 7-12: FP2;
	BTS Smart Capture			IORgait [47]			13-17: EMG
UMG	Qualysis		240	modified version	-	720	1-6: FP1; 8-13: FP2
	Qualysis Track Manager (QTM)			of [48]			
GU	Vicon		200	10 Points Cluster [49]	Aurion Zero Wire	1000	1-6: FP1; 7-12: FP2;
	Vicon Nexus						29-44:EMG;
							13-28, 45-52:Biodex
UWA	Vicon		250	UWA full-body [50]	Noraxon 2400T G2	2000	1-6: FP1; 7-12: FP2;
	Vicon Nexus						13-28: EMG

Four institutions are involved: Department of Information Engineering, University of Padova, Italy (UNIPD), Department of Neurorehabilitation Engineering, Georg August University in Gottingen, Germany (UMG), Centre of Musculoskeletal Research, Griffith University, Gold Coast, Australia (GU), and School of Sport Science, Exercise and Health, University of Western Australia, Perth, Australia (UWA)

**Fig. 5:**
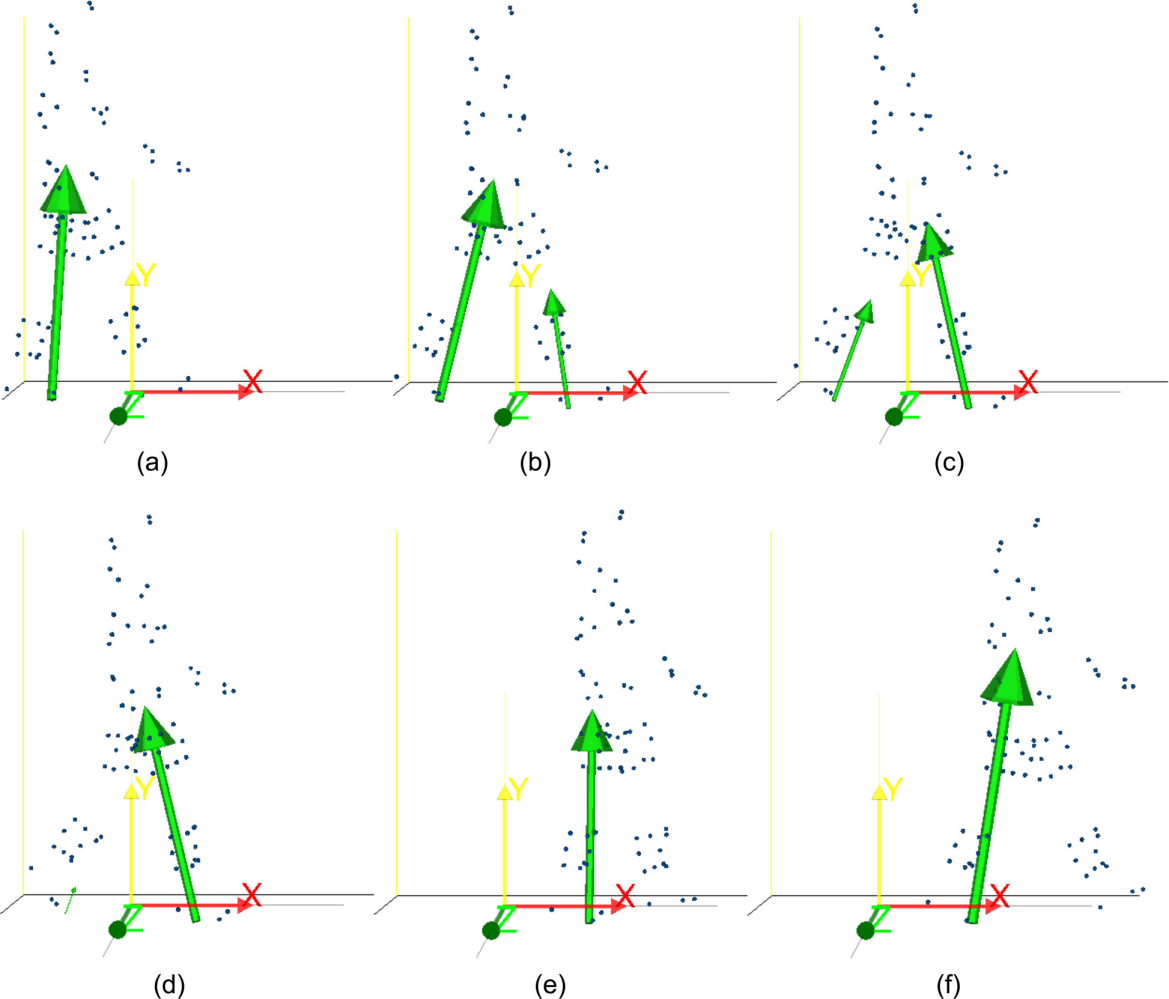
Gait cycle in OpenSim. Example of.trc and.mot files generated using MOtoNMS and loaded in OpenSim. The sequence (**a**-**f**) reproduces a gait cycle on the laboratory force platforms

**Fig. 6:**
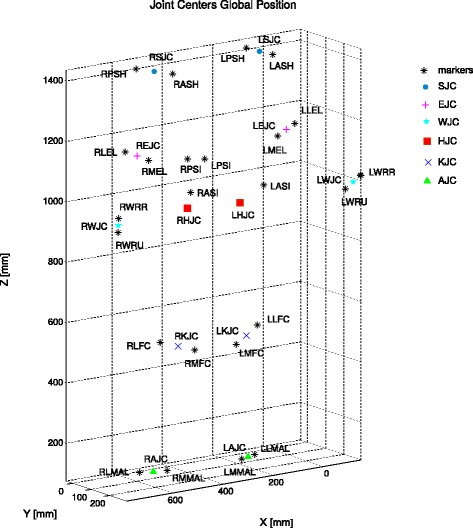
Joint centers. A 3D view of hip (*HJC*), knee (*KJC*), ankle (*AJC*), elbow (*EJC*), shoulder (*SJC*) and wrist (*WJC*) joint centers and markers used for their computation

**Fig. 7:**
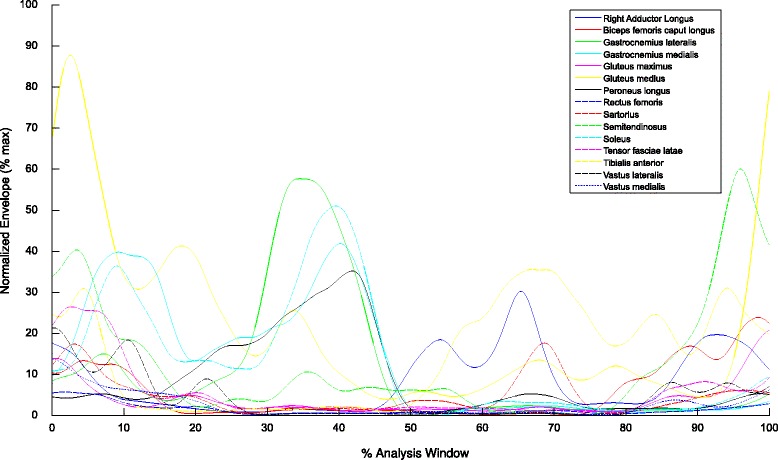
Normalized EMG linear envelopes. Normalized EMG linear envelopes versus the percentage of the analysis window selected for the elaboration. All muscles of a single acquisition are grouped together to provide a global picture of the output of the EMG processing step

**Fig. 8:**
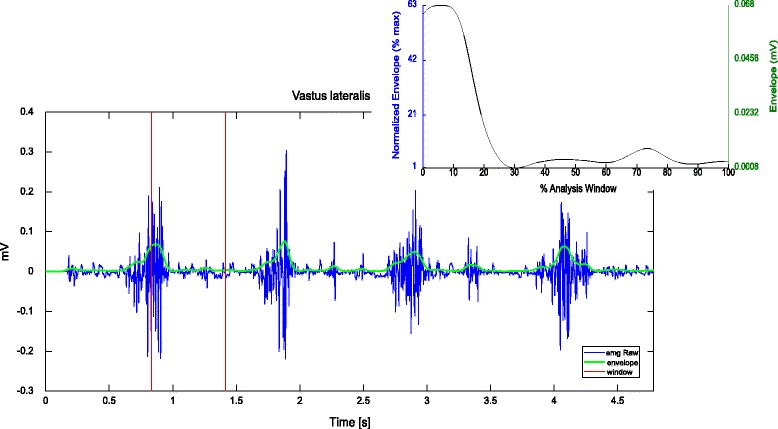
Example of output EMG plots. The main plot shows raw EMG (*blue*) for an overall trial, together with the computed envelope (*green*) and the selected analysis window (*red*). An example of plot of an envelope within the analysis window is reported in the smaller picture. Two measurement scales are visible in the graph: the normalized one (*blue, on the left*), and the voltage from the acquisition device (*green, on the right*)

Experimental data were collected from four healthy subjects, one for each institution, who gave their informed consent. MOtoNMS was used to elaborate the collected movement trials and produce the following outputs: (1).trc and.mot files for OpenSim (Fig. 5), (2) joint centers for hip, knee, and ankle and, depending on data availability, also wrist, elbow, and shoulder (Fig. 6), (3) normalized EMG linear envelopes (Fig. 7), and (4) plots of processed data (Fig. 8).

Tests aimed at proving the correctness of execution on different combinations of configuration options, i.e., the definition of the analysis window, the cut-off frequencies for filtering, number and combination of trials to be elaborated and different sets of trials for the computation of the maximum EMG peak.

To illustrate MOtoNMS capabilities, a selection of the collected trials and examples of obtained results with the corresponding configuration files are freely available for download [37]. Three elaborations for the dynamic trials and one for the static acquisitions are included for each data set. Resulting.trc and.mot files can be directly loaded in OpenSim and used to visualize the processed data. The full MATLAB source code of MOtoNMS [27] with the User Manual [36] is also available to allow reproducibility of results and additional testing.

Results show that, despite the differences in instruments, configurations, and protocols (Tables 1 and 2), MOtoNMS succeeded in processing data in a consistent and repeatable way, based on the parameters selected in the user-defined configuration files.

**Table 2:** FPs characteristics of the laboratories testing MOtoNMS

Institution	Num	Brand and Model	Type	Sizes (mm)	Position along the walkway
UNIPD	2	Bertec	1	400×600	
		4060-08-1000		400×600	
UMG	2	Bertec	4	400×600	
		4060-07-1000		400×600	
GU	2	Kistler 9287B	2	900×600	
				800×600	
UWA	2	AMTI BP12001200	2	1200×1200	
		Kistler 9281C		400×600	

Different FP types require different procedures for plate moments and CoP computation. Force platform of type 3 is not available in the laboratories, but it is implemented in the toolbox and it has been tested by another institution

## Discussion and conclusions

MOtoNMS enables processing motion data collected with different instruments and procedures, and generates inputs for neuromusculoskeletal modeling software. Marker trajectories, GRFs, and joint centers are processed and saved using OpenSim file formats [9], while normalized EMG linear envelopes are exported by default to the OpenSim motion file format (.mot), compatible also with CEINMS [30].

MOtoNMS has been designed to be flexible and highly configurable, to satisfy the requests of different research groups without the need of accessing and modifying the code. Indeed, processing properties (i.e., selected trials, cut-off frequencies, data analysis window, markers list, joint centers of interest, …) can be selected directly from user-friendly graphical interfaces and stored, together with the laboratory arrangements, in configuration files. In addition, processed data, along with the configuration and processing log files, are automatically organized in output directories with a uniquely defined structure. This becomes an essential feature for information retrieval and when results are shared among different research teams, especially if large amount of data are involved. Finally, MOtoNMS has been developed in MATLAB for its large diffusion in biomechanics research, and works on the most diffused operating systems (Windows, Linux, and Mac OS X).

Currently available alternatives to MOtoNMS do not provide complete solutions that generalize across laboratories. Lee S. and Son J. proposed a toolbox that converts motion data in OpenSim inputs [38], however it is limited to VICON systems only. Other MATLAB functions with a broader applicability are available on the SimTK.org website [39, 40]. While they implement several tasks, they are not connected in a well-structured instrument able to fully process data in a single procedure [41, 42]. The users are required to go through a sequence of MATLAB functions and often to adapt the code to their own laboratory configuration and experimental protocols. Tim Dorn provides a complete tool with the C3D Extraction Toolbox [43]. However, support and testing of different laboratory setup is limited to specific instrumentation types (e.g., assumption of AMTI force plates). Finally, none of these solutions provide a tool to process the recorded data supplying filtering blocks, several methods for the analysis windows selection, computation of joint centers, EMG linear envelopes and maximum EMG peaks from selected trials for normalization, and graphical interfaces.

Results showed that MOtoNMS could instead be used to process data from laboratories of four institutions (Table 1) with three different motion capture systems (i.e., Vicon, BTS, Qualisys), EMG units (Noraxon, BTS, and Zerowire), as well as GRF data generated by four different force plate types (e.g., types 1 to 4 by Bertec, AMTI, and Krisler, Table 2). This makes MOtoNMS the first toolbox that allows users to easily configure the processing of motion data from laboratories with different instruments, software, protocols, and methodologies, and export data processed for musculoskeletal applications. MOtoNMS currently supports OpenSim and CEINMS file formats. Nevertheless, its modular design supports the integration of additional blocks for the generation of output files required by other musculoskeletal applications.

MOtoNMS is an ongoing software with a dynamic cycle of development, aimed at extending its features. Additional methods for joint centers computation, e.g. based on functional movements, may be included in a near future. Customizable algorithms for a better control in the computation of EMG maximum and average could also be introduced. We are also planning to distribute a database of configuration files for the most popular acquisition protocols [44–46]. In addition, we will provide a standalone application of MOtoNMS using the MATLAB Runtime Compiler that will allow the use of the software in the contexts, such as the clinical one, where the diffusion of MATLAB could be limited.

MOtoNMS is released under GNU GPL license and latest versions of the toolbox are constantly uploaded on the project page at the SimTK.org website [37], together with up-to-date documentation and a set of testing data. The GitHub repository of the project traces changes in the development of the software and aims at encouraging contributions to extend MOtoNMS capabilities from other users [27].

The authors hope that MOtoNMS will be useful to the research community, reducing the gap between experimental motion data and neuromusculoskeletal simulation software, and uniforming data processing methods across laboratories. Moreover, reduction of processing time and the intuitive graphical user interfaces may facilitate the translation of neuromusculoskeletal modeling and simulation to daily and clinical practice.

## Availability and requirements

**Project name:** MOtoNMS**Project home page:**https://simtk.org/home/motonms/**Repository:**https://github.com/RehabEngGroup/MOtoNMS (public GIT repository)**DOI:** 10.5281/zenodo.18690**Test Data:**https://simtk.org/home/motonms/**Documentation:**http://rehabenggroup.github.io/MOtoNMS/ [User Manual]**Operating system(s):** Platform independent**Programming language:** MATLAB**Other requirements:** C3Dserver (http://www.c3dserver.com/) or Biomechanical Toolkit (BTK, https://code.google.com/p/b-tk/)**License:** GNU General Public License v3**Any restrictions to use by non-academics:** None

## Abbreviations

BTK: Biomechanical Toolkit
C3D: Coordinate 3D
CoP: Center of Pressure
EMG: Electromyography
FP: Force Platform
GPL: GNU General Public License
GRFs: Foot Ground Reaction Forces
XML: Extensibile Markup Language
XSD: XML Schema Definition
